# Supplementary material for: Single Cell Transcriptomic and Chromatin Profiles Suggest Layer Vb Is the Only Layer With Shared Excitatory Cell Types in the Medial and Lateral Entorhinal Cortex
Source: Front Neural Circuits. 2022 Jan 26;15:806154. doi: 10.3389/fncir.2021.806154 (PMC8826650; doi:10.3389/fncir.2021.806154)
Supplement: Supplementary file 14 [file Data_Sheet_1.ZIP › 5/knownResults.html]

5 - Homer Known Motif Enrichment Results


# Homer Known Motif Enrichment Results (5)

Homer *de novo* Motif Results  
Gene Ontology Enrichment Results  
Known Motif Enrichment Results (txt file)  
Total Target Sequences = 1583, Total Background Sequences = 45890

|  |  |  |  |  |  |  |  |  |  |  |  |
| --- | --- | --- | --- | --- | --- | --- | --- | --- | --- | --- | --- |
| Rank | Motif | Name | P-value | log P-pvalue | q-value (Benjamini) | # Target Sequences with Motif | % of Targets Sequences with Motif | # Background Sequences with Motif | % of Background Sequences with Motif | Motif File | SVG |
| 1 | C G A T T G C A T G C A G A T C C G T A A C T G T G A C G A C T C A T G A C T G | Tcf21(bHLH)/ArterySmoothMuscle-Tcf21-ChIP-Seq(GSE61369)/Homer | 1e-171 | -3.958e+02 | 0.0000 | 620.0 | 39.17% | 5350.8 | 11.66% | motif file (matrix) | svg |
| 2 | T G A C A G T C C G T A A C T G G T A C A C G T A C T G A C G T G A C T G A T C | Twist2(bHLH)/Myoblast-Twist2.Ty1-ChIP-Seq(GSE127998)/Homer | 1e-143 | -3.306e+02 | 0.0000 | 894.0 | 56.48% | 11948.0 | 26.03% | motif file (matrix) | svg |
| 3 | C T A G T C G A T G A C A G T C C G T A A C T G G T A C A C G T A C T G A C T G | BHLHA15(bHLH)/NIH3T3-BHLHB8.HA-ChIP-Seq(GSE119782)/Homer | 1e-137 | -3.168e+02 | 0.0000 | 754.0 | 47.63% | 9011.0 | 19.63% | motif file (matrix) | svg |
| 4 | T C G A A G T C C G T A A T C G A T G C C G A T A C T G A G T C A G C T A C T G | Tcf12(bHLH)/GM12878-Tcf12-ChIP-Seq(GSE32465)/Homer | 1e-129 | -2.974e+02 | 0.0000 | 535.0 | 33.80% | 5007.0 | 10.91% | motif file (matrix) | svg |
| 5 | G T C A T G C A G C T A A G T C C G T A A C T G T G A C G C A T T C A G C A G T | Ap4(bHLH)/AML-Tfap4-ChIP-Seq(GSE45738)/Homer | 1e-125 | -2.900e+02 | 0.0000 | 654.0 | 41.31% | 7356.8 | 16.03% | motif file (matrix) | svg |
| 6 | C T A G A G T C T A C G T A C G T G A C C G T A A C T G T A G C G C A T C A T G A T G C A G C T | Ascl1(bHLH)/NeuralTubes-Ascl1-ChIP-Seq(GSE55840)/Homer | 1e-107 | -2.473e+02 | 0.0000 | 680.0 | 42.96% | 8616.5 | 18.77% | motif file (matrix) | svg |
| 7 | C T G A T C A G A G T C C G T A A T C G A T G C C G A T A C T G A G T C G A C T A T C G A G T C | MyoD(bHLH)/Myotube-MyoD-ChIP-Seq(GSE21614)/Homer | 1e-105 | -2.423e+02 | 0.0000 | 449.0 | 28.36% | 4196.3 | 9.14% | motif file (matrix) | svg |
| 8 | T C G A T C G A A G T C C G T A C T A G T A G C A C G T A C T G | MyoG(bHLH)/C2C12-MyoG-ChIP-Seq(GSE36024)/Homer | 1e-103 | -2.390e+02 | 0.0000 | 531.0 | 33.54% | 5727.0 | 12.48% | motif file (matrix) | svg |
| 9 | A C G T T C G A T C G A A G T C G T C A T A C G A T G C A C G T A C T G A G C T | Myf5(bHLH)/GM-Myf5-ChIP-Seq(GSE24852)/Homer | 1e-102 | -2.365e+02 | 0.0000 | 430.0 | 27.16% | 3945.6 | 8.60% | motif file (matrix) | svg |
| 10 | T A C G T G C A A G T C C G T A A C G T T G A C A C G T A C T G A C T G G C A T | TCF4(bHLH)/SHSY5Y-TCF4-ChIP-Seq(GSE96915)/Homer | 1e-101 | -2.330e+02 | 0.0000 | 746.0 | 47.13% | 10369.1 | 22.59% | motif file (matrix) | svg |
| 11 | T G A C A T G C C G T A A T C G A T G C C A G T C A T G A C T G A G T C G T A C | HEB(bHLH)/mES-Heb-ChIP-Seq(GSE53233)/Homer | 1e-100 | -2.311e+02 | 0.0000 | 742.0 | 46.87% | 10315.6 | 22.48% | motif file (matrix) | svg |
| 12 | T C G A A G T C C G T A A T C G T A G C A C G T A C T G A G C T A C G T A G T C | Ptf1a(bHLH)/Panc1-Ptf1a-ChIP-Seq(GSE47459)/Homer | 1e-98 | -2.258e+02 | 0.0000 | 957.0 | 60.45% | 15800.2 | 34.42% | motif file (matrix) | svg |
| 13 | T G C A C T G A A T G C G T C A A C G T A T G C A C G T A C T G A C T G T G C A | ZBTB18(Zf)/HEK293-ZBTB18.GFP-ChIP-Seq(GSE58341)/Homer | 1e-96 | -2.229e+02 | 0.0000 | 374.0 | 23.63% | 3184.3 | 6.94% | motif file (matrix) | svg |
| 14 | T C A G A C G T T C G A T A G C A G T C C G T A A C T G G T A C A C G T A C T G A T C G A G T C | Atoh1(bHLH)/Cerebellum-Atoh1-ChIP-Seq(GSE22111)/Homer | 1e-95 | -2.195e+02 | 0.0000 | 561.0 | 35.44% | 6626.1 | 14.44% | motif file (matrix) | svg |
| 15 | T A C G T A C G C T A G T C A G A G T C C G T A A T C G A T G C A C G T A C T G A G T C G A C T | Ascl2(bHLH)/ESC-Ascl2-ChIP-Seq(GSE97712)/Homer | 1e-91 | -2.116e+02 | 0.0000 | 521.0 | 32.91% | 5964.9 | 13.00% | motif file (matrix) | svg |
| 16 | C G T A T G A C T C G A A G T C C G T A A T C G A T G C A C G T A C T G A G T C | E2A(bHLH)/proBcell-E2A-ChIP-Seq(GSE21978)/Homer | 1e-90 | -2.092e+02 | 0.0000 | 603.0 | 38.09% | 7677.4 | 16.73% | motif file (matrix) | svg |
| 17 | T C G A T G A C G T A C C G T A C A G T T G A C A C G T A C T G A G C T A G C T | NeuroG2(bHLH)/Fibroblast-NeuroG2-ChIP-Seq(GSE75910)/Homer | 1e-82 | -1.893e+02 | 0.0000 | 705.0 | 44.54% | 10369.7 | 22.59% | motif file (matrix) | svg |
| 18 | C T G A T C A G G T A C G C T A A C T G T G A C G C A T C A T G | SCL(bHLH)/HPC7-Scl-ChIP-Seq(GSE13511)/Homer | 1e-79 | -1.829e+02 | 0.0000 | 1287.0 | 81.30% | 27099.5 | 59.04% | motif file (matrix) | svg |
| 19 | C T G A T G A C T G A C C G T A A C G T T G A C A G C T C T A G A C G T G A C T | Olig2(bHLH)/Neuron-Olig2-ChIP-Seq(GSE30882)/Homer | 1e-50 | -1.160e+02 | 0.0000 | 758.0 | 47.88% | 13712.3 | 29.88% | motif file (matrix) | svg |
| 20 | G C A T C G T A C G A T A C T G A G T C G C T A C T G A C G T A C A G T A C T G C G T A T C A G | Oct6(POU,Homeobox)/NPC-Pou3f1-ChIP-Seq(GSE35496)/Homer | 1e-48 | -1.126e+02 | 0.0000 | 331.0 | 20.91% | 4015.4 | 8.75% | motif file (matrix) | svg |
| 21 | G A C T C G T A A C G T A C T G A G T C C G T A C T G A C G T A C A G T A C G T G T C A T C A G | Brn1(POU,Homeobox)/NPC-Brn1-ChIP-Seq(GSE35496)/Homer | 1e-44 | -1.034e+02 | 0.0000 | 262.0 | 16.55% | 2888.7 | 6.29% | motif file (matrix) | svg |
| 22 | C G T A A C G T A G C T C G A T C T A G G T A C C G T A A G C T C G T A G C T A | Oct4(POU,Homeobox)/mES-Oct4-ChIP-Seq(GSE11431)/Homer | 1e-40 | -9.373e+01 | 0.0000 | 332.0 | 20.97% | 4422.1 | 9.63% | motif file (matrix) | svg |
| 23 | T C A G T G A C G T A C C G T A A C G T T G A C A C G T T C A G A G C T G A C T | NeuroD1(bHLH)/Islet-NeuroD1-ChIP-Seq(GSE30298)/Homer | 1e-39 | -9.164e+01 | 0.0000 | 349.0 | 22.05% | 4820.0 | 10.50% | motif file (matrix) | svg |
| 24 | A C T G G T C A A C G T G C A T C G A T T C A G G T A C G T C A A C G T C T G A | Oct11(POU,Homeobox)/NCIH1048-POU2F3-ChIP-seq(GSE115123)/Homer | 1e-37 | -8.561e+01 | 0.0000 | 250.0 | 15.79% | 2982.2 | 6.50% | motif file (matrix) | svg |
| 25 | G T C A G C A T G C T A C A G T C T A G G A T C C G T A C T G A C G T A C G A T | Oct2(POU,Homeobox)/Bcell-Oct2-ChIP-Seq(GSE21512)/Homer | 1e-33 | -7.609e+01 | 0.0000 | 242.0 | 15.29% | 3016.4 | 6.57% | motif file (matrix) | svg |
| 26 | G A C T C G A T C T G A G T C A G A C T C G A T T C G A C G T A G C T A G C T A T G A C G T A C C G T A A C T G T G C A C G A T A C T G A C G T | Pitx1:Ebox(Homeobox,bHLH)/Hindlimb-Pitx1-ChIP-Seq(GSE41591)/Homer | 1e-25 | -5.810e+01 | 0.0000 | 143.0 | 9.03% | 1528.7 | 3.33% | motif file (matrix) | svg |
| 27 | G C T A G C A T G A C T G C A T T C A G G T A C G C T A G C A T C T G A G C T A T A G C G C T A C T G A C G A T C T A G | OCT4-SOX2-TCF-NANOG(POU,Homeobox,HMG)/mES-Oct4-ChIP-Seq(GSE11431)/Homer | 1e-23 | -5.393e+01 | 0.0000 | 152.0 | 9.60% | 1760.9 | 3.84% | motif file (matrix) | svg |
| 28 | G C T A A C G T C T A G G T A C G C T A G A C T C T G A G C A T C A T G G A T C | Pit1(Homeobox)/GCrat-Pit1-ChIP-Seq(GSE58009)/Homer | 1e-19 | -4.415e+01 | 0.0000 | 498.0 | 31.46% | 9924.9 | 21.62% | motif file (matrix) | svg |
| 29 | G C T A A G C T T A C G G T C A C T G A C G A T C T G A G C A T C A G T A G T C | Brn2(POU,Homeobox)/NPC-Brn2-ChIP-Seq(GSE35496)/Homer | 1e-18 | -4.319e+01 | 0.0000 | 92.0 | 5.81% | 897.1 | 1.95% | motif file (matrix) | svg |
| 30 | G C T A A C G T C T A G G T C A C G T A A C G T C G T A C G A T C A G T A G T C C G T A A C G T C T A G C T G A A T C G | OCT:OCT(POU,Homeobox)/NPC-Brn1-ChIP-Seq(GSE35496)/Homer | 1e-13 | -3.028e+01 | 0.0000 | 25.0 | 1.58% | 101.4 | 0.22% | motif file (matrix) | svg |
| 31 | G A C T C T A G G A T C C A G T A C T G C T G A A T G C G C A T A T G C C T G A | MafA(bZIP)/Islet-MafA-ChIP-Seq(GSE30298)/Homer | 1e-12 | -2.794e+01 | 0.0000 | 290.0 | 18.32% | 5562.8 | 12.12% | motif file (matrix) | svg |
| 32 | G T C A A C G T C T A G G T C A C G T A G C A T C G T A C G T A G C A T C A G T A G T C C G T A C A G T C T A G C T G A | OCT:OCT(POU,Homeobox,IR1)/NPC-Brn2-ChIP-Seq(GSE35496)/Homer | 1e-11 | -2.545e+01 | 0.0000 | 27.0 | 1.71% | 149.8 | 0.33% | motif file (matrix) | svg |
| 33 | C A T G A G T C G A C T C G T A C G A T G C A T G A C T G C A T C G A T C T A G C A T G T G A C | Mef2b(MADS)/HEK293-Mef2b.V5-ChIP-Seq(GSE67450)/Homer | 1e-10 | -2.464e+01 | 0.0000 | 314.0 | 19.84% | 6324.7 | 13.78% | motif file (matrix) | svg |
| 34 | T G C A C G T A G T C A A G C T A G T C G C T A T A G C C G A T C T A G G A T C | Gfi1b(Zf)/HPC7-Gfi1b-ChIP-Seq(GSE22178)/Homer | 1e-10 | -2.443e+01 | 0.0000 | 249.0 | 15.73% | 4745.6 | 10.34% | motif file (matrix) | svg |
| 35 | C G T A G A C T C G A T A T C G G T A C G C A T C A T G C G T A T A C G G C A T G T A C C G T A C A T G A T G C G C T A C T A G G C A T G C A T G C A T G A C T | MafB(bZIP)/BMM-Mafb-ChIP-Seq(GSE75722)/Homer | 1e-9 | -2.285e+01 | 0.0000 | 184.0 | 11.62% | 3285.7 | 7.16% | motif file (matrix) | svg |
| 36 | C G T A A C G T A C T G G T A C C G T A A C G T C G T A C G T A A C G T A C T G A G T C C G T A A C G T C T G A G C A T | OCT:OCT-short(POU,Homeobox)/NPC-OCT6-ChIP-Seq(GSE43916)/Homer | 1e-9 | -2.278e+01 | 0.0000 | 315.0 | 19.90% | 6456.1 | 14.07% | motif file (matrix) | svg |
| 37 | T C A G G A C T C A G T C T G A A G C T C T A G G A C T T G C A C T G A A G T C | HLF(bZIP)/HSC-HLF.Flag-ChIP-Seq(GSE69817)/Homer | 1e-9 | -2.160e+01 | 0.0000 | 311.0 | 19.65% | 6426.6 | 14.00% | motif file (matrix) | svg |
| 38 | C A T G G T A C G A C T G C T A C G T A C G T A C G T A G C T A G A C T C T G A T C A G G T A C | Mef2c(MADS)/GM12878-Mef2c-ChIP-Seq(GSE32465)/Homer | 1e-9 | -2.082e+01 | 0.0000 | 189.0 | 11.94% | 3491.2 | 7.61% | motif file (matrix) | svg |
| 39 | G T A C G A C T C G T A C T G A T C G A C G T A G C T A C A G T C T G A T A C G | Mef2a(MADS)/HL1-Mef2a.biotin-ChIP-Seq(GSE21529)/Homer | 1e-8 | -2.018e+01 | 0.0000 | 167.0 | 10.55% | 3007.9 | 6.55% | motif file (matrix) | svg |
| 40 | T C G A A C G T C A T G G C T A T A G C C G A T G T A C G C T A A C G T A T G C | AP-1(bZIP)/ThioMac-PU.1-ChIP-Seq(GSE21512)/Homer | 1e-8 | -1.901e+01 | 0.0000 | 232.0 | 14.66% | 4615.7 | 10.06% | motif file (matrix) | svg |
| 41 | C A T G A G T C A G C T C G T A C G A T C G A T G C A T G C A T C G A T C T G A C A T G T G A C | Mef2d(MADS)/Retina-Mef2d-ChIP-Seq(GSE61391)/Homer | 1e-7 | -1.824e+01 | 0.0000 | 87.0 | 5.50% | 1309.3 | 2.85% | motif file (matrix) | svg |
| 42 | A C T G C A T G T C G A A C G T A C T G C G T A A T C G A C G T G T A C C G T A G A C T A G T C | Fos(bZIP)/TSC-Fos-ChIP-Seq(GSE110950)/Homer | 1e-7 | -1.820e+01 | 0.0000 | 192.0 | 12.13% | 3689.1 | 8.04% | motif file (matrix) | svg |
| 43 | C T A G T A C G G A T C G T A C G C T A A G C T A G C T G T C A T C G A T A G C | Nanog(Homeobox)/mES-Nanog-ChIP-Seq(GSE11724)/Homer | 1e-7 | -1.761e+01 | 0.0000 | 1090.0 | 68.86% | 28569.7 | 62.25% | motif file (matrix) | svg |
| 44 | C T A G T C G A A C G T A C T G C G T A A T G C A C G T G T A C C G T A A G C T G A T C G T A C | Atf3(bZIP)/GBM-ATF3-ChIP-Seq(GSE33912)/Homer | 1e-7 | -1.758e+01 | 0.0000 | 209.0 | 13.20% | 4133.7 | 9.01% | motif file (matrix) | svg |
| 45 | A C T G C T A G T C G A C G A T C A T G G C T A A T C G C G A T G T A C G C T A A G C T G T A C | Fra1(bZIP)/BT549-Fra1-ChIP-Seq(GSE46166)/Homer | 1e-7 | -1.728e+01 | 0.0000 | 180.0 | 11.37% | 3449.9 | 7.52% | motif file (matrix) | svg |
| 46 | T A G C T C A G C A T G G C A T A G C T C G A T A T G C C G T A C G T A G T C A | CHR(?)/Hela-CellCycle-Expression/Homer | 1e-6 | -1.598e+01 | 0.0000 | 248.0 | 15.67% | 5194.9 | 11.32% | motif file (matrix) | svg |
| 47 | C A G T T G C A A C G T A C T G C G T A A T C G C G A T T G A C C G T A A C G T | BATF(bZIP)/Th17-BATF-ChIP-Seq(GSE39756)/Homer | 1e-6 | -1.569e+01 | 0.0000 | 206.0 | 13.01% | 4167.5 | 9.08% | motif file (matrix) | svg |
| 48 | C T A G T C G A C G A T C T A G G C A T C A G T C T A G G A T C C G T A G T C A | CEBP:AP1(bZIP)/ThioMac-CEBPb-ChIP-Seq(GSE21512)/Homer | 1e-6 | -1.543e+01 | 0.0000 | 250.0 | 15.79% | 5281.8 | 11.51% | motif file (matrix) | svg |
| 49 | A G T C A C G T A C T G A G C T A C G T A C G T G T C A A G T C | Foxo1(Forkhead)/RAW-Foxo1-ChIP-Seq(Fan\_et\_al.)/Homer | 1e-6 | -1.506e+01 | 0.0000 | 546.0 | 34.49% | 13163.2 | 28.68% | motif file (matrix) | svg |
| 50 | T C G A A C G T A C G T C T G A G A T C T C A G G A C T G T C A C G T A A G C T G T C A C T A G A G C T A C G T T C G A | NFIL3(bZIP)/HepG2-NFIL3-ChIP-Seq(Encode)/Homer | 1e-6 | -1.441e+01 | 0.0000 | 216.0 | 13.64% | 4494.6 | 9.79% | motif file (matrix) | svg |
| 51 | C G T A T A G C T A G C T G C A A C T G C T A G C G T A C G T A T C A G G A C T | EHF(ETS)/LoVo-EHF-ChIP-Seq(GSE49402)/Homer | 1e-6 | -1.415e+01 | 0.0000 | 371.0 | 23.44% | 8517.9 | 18.56% | motif file (matrix) | svg |
| 52 | T C A G C T A G A T G C G A C T T G C A C G T A A G C T A C G T C T G A T A C G | En1(Homeobox)/SUM149-EN1-ChIP-Seq(GSE120957)/Homer | 1e-5 | -1.365e+01 | 0.0000 | 598.0 | 37.78% | 14748.8 | 32.13% | motif file (matrix) | svg |
| 53 | T G C A A G C T C A T G C G T A A G C T A C T G G A T C G T C A C G T A A G C T | Atf4(bZIP)/MEF-Atf4-ChIP-Seq(GSE35681)/Homer | 1e-5 | -1.357e+01 | 0.0000 | 105.0 | 6.63% | 1869.1 | 4.07% | motif file (matrix) | svg |
| 54 | A T G C A G T C C T G A A G T C C G A T A C G T A G T C A G T C A C G T A T C G G A C T A C G T | Etv2(ETS)/ES-ER71-ChIP-Seq(GSE59402)/Homer | 1e-5 | -1.327e+01 | 0.0000 | 259.0 | 16.36% | 5663.5 | 12.34% | motif file (matrix) | svg |
| 55 | C A T G C T A G T C G A A C G T A C T G C G T A T A G C C G A T T G A C C G T A A G C T G A T C | Fra2(bZIP)/Striatum-Fra2-ChIP-Seq(GSE43429)/Homer | 1e-5 | -1.257e+01 | 0.0000 | 142.0 | 8.97% | 2787.2 | 6.07% | motif file (matrix) | svg |
| 56 | T C G A T A G C G T C A A C T G A C T G C G T A C G T A C T A G A G C T T C A G | ERG(ETS)/VCaP-ERG-ChIP-Seq(GSE14097)/Homer | 1e-5 | -1.238e+01 | 0.0000 | 415.0 | 26.22% | 9859.6 | 21.48% | motif file (matrix) | svg |
| 57 | C A T G T A C G T A G C G A T C G A T C A T G C G T A C G A C T T C A G A T G C C G A T A T C G C A G T A C T G G T A C | Zic3(Zf)/mES-Zic3-ChIP-Seq(GSE37889)/Homer | 1e-5 | -1.171e+01 | 0.0001 | 124.0 | 7.83% | 2402.8 | 5.24% | motif file (matrix) | svg |
| 58 | G T A C C G T A C G T A T A C G G C A T G T A C C G T A C A T G A G T C C G T A C G T A C G A T G C A T G C A T G A C T | MafF(bZIP)/HepG2-MafF-ChIP-Seq(GSE31477)/Homer | 1e-5 | -1.157e+01 | 0.0001 | 124.0 | 7.83% | 2409.1 | 5.25% | motif file (matrix) | svg |
| 59 | G A C T C A G T G A T C G A T C A C G T G A T C C T G A T A C G C G T A G T C A | STAT6(Stat)/Macrophage-Stat6-ChIP-Seq(GSE38377)/Homer | 1e-4 | -1.118e+01 | 0.0001 | 188.0 | 11.88% | 4009.8 | 8.74% | motif file (matrix) | svg |
| 60 | A G T C C T G A A G T C C G A T C A G T G A T C A T G C A C T G A T C G G A C T | Fli1(ETS)/CD8-FLI-ChIP-Seq(GSE20898)/Homer | 1e-4 | -1.102e+01 | 0.0001 | 271.0 | 17.12% | 6155.2 | 13.41% | motif file (matrix) | svg |
| 61 | A T C G A T G C A G T C T A G C G A C T T C G A G C T A G C A T A G C T C T G A | DLX1(Homeobox)/BasalGanglia-Dlx1-ChIP-seq(GSE124936)/Homer | 1e-4 | -1.095e+01 | 0.0001 | 462.0 | 29.19% | 11288.8 | 24.60% | motif file (matrix) | svg |
| 62 | C T G A A G C T A C G T A C G T A G T C G A C T G A C T C T G A C T G A C T A G C G T A C G T A | STAT6(Stat)/CD4-Stat6-ChIP-Seq(GSE22104)/Homer | 1e-4 | -1.079e+01 | 0.0001 | 186.0 | 11.75% | 3986.9 | 8.69% | motif file (matrix) | svg |
| 63 | C T A G T C G A A C G T A C T G C G T A T A G C C G A T G T A C C G T A A G C T G A T C G T A C | Jun-AP1(bZIP)/K562-cJun-ChIP-Seq(GSE31477)/Homer | 1e-4 | -1.057e+01 | 0.0002 | 71.0 | 4.49% | 1225.3 | 2.67% | motif file (matrix) | svg |
| 64 | C T A G T C G A G C A T C A T G G C T A T A G C C G A T G T A C C T G A A G C T | JunB(bZIP)/DendriticCells-Junb-ChIP-Seq(GSE36099)/Homer | 1e-4 | -1.046e+01 | 0.0002 | 161.0 | 10.17% | 3381.5 | 7.37% | motif file (matrix) | svg |
| 65 | C T A G T C G A C G A T A C T G C G T A T A C G A G C T T G A C G C T A A C G T G A T C T A G C | Fosl2(bZIP)/3T3L1-Fosl2-ChIP-Seq(GSE56872)/Homer | 1e-4 | -1.028e+01 | 0.0002 | 94.0 | 5.94% | 1765.6 | 3.85% | motif file (matrix) | svg |
| 66 | C G T A C G T A C G T A G C A T G C A T T A C G G T A C G A C T A C T G C G T A A T C G A C G T G T A C C G T A A G C T | Bach1(bZIP)/K562-Bach1-ChIP-Seq(GSE31477)/Homer | 1e-4 | -1.004e+01 | 0.0003 | 29.0 | 1.83% | 363.7 | 0.79% | motif file (matrix) | svg |
| 67 | T C G A A G C T A C G T A C G T A G T C A G T C A C G T A T C G G A C T A T C G | EWS:ERG-fusion(ETS)/CADO\_ES1-EWS:ERG-ChIP-Seq(SRA014231)/Homer | 1e-4 | -9.976e+00 | 0.0003 | 228.0 | 14.40% | 5125.9 | 11.17% | motif file (matrix) | svg |
| 68 | G C T A C G A T C T A G G T A C G C T A C G A T C G T A G C T A C G A T C A G T G A T C G C T A | Pit1+1bp(Homeobox)/GCrat-Pit1-ChIP-Seq(GSE58009)/Homer | 1e-4 | -9.966e+00 | 0.0003 | 159.0 | 10.04% | 3365.6 | 7.33% | motif file (matrix) | svg |
| 69 | T G A C G C T A T G A C C G T A T C A G G A T C C G T A C A T G C A T G C T A G C T A G C T A G | Unknown-ESC-element(?)/mES-Nanog-ChIP-Seq(GSE11724)/Homer | 1e-4 | -9.595e+00 | 0.0004 | 143.0 | 9.03% | 2991.9 | 6.52% | motif file (matrix) | svg |
| 70 | A T G C T A C G A G C T T G C A C G T A C G A T A C G T C T G A | DLX5(Homeobox)/BasalGanglia-Dlx5-ChIP-seq(GSE124936)/Homer | 1e-4 | -9.264e+00 | 0.0006 | 277.0 | 17.50% | 6475.5 | 14.11% | motif file (matrix) | svg |
| 71 | T G A C G T A C C G T A A C T G T G A C C G A T A C T G A T C G A G C T T A C G T C G A T A G C G T A C C G T A A T C G T G A C G C A T A C T G A C T G A T G C | Twist(bHLH)/HMLE-TWIST1-ChIP-Seq(Chang\_et\_al)/Homer | 1e-4 | -9.249e+00 | 0.0006 | 45.0 | 2.84% | 705.9 | 1.54% | motif file (matrix) | svg |
| 72 | C T A G A C T G C T A G T C A G T C A G T A C G C T A G A C T G | Maz(Zf)/HepG2-Maz-ChIP-Seq(GSE31477)/Homer | 1e-3 | -9.041e+00 | 0.0007 | 210.0 | 13.27% | 4738.2 | 10.32% | motif file (matrix) | svg |
| 73 | T C G A A C G T A C T G C T G A A G T C T C A G A G C T G T A C C G T A A G C T G A T C T C G A | JunD(bZIP)/K562-JunD-ChIP-Seq/Homer | 1e-3 | -8.761e+00 | 0.0009 | 32.0 | 2.02% | 451.9 | 0.98% | motif file (matrix) | svg |
| 74 | C G T A T G A C T A G C T G C A A C T G A C T G C G T A C G T A T C A G G A C T | ELF3(ETS)/PDAC-ELF3-ChIP-Seq(GSE64557)/Homer | 1e-3 | -8.380e+00 | 0.0014 | 215.0 | 13.58% | 4927.4 | 10.74% | motif file (matrix) | svg |
| 75 | A C T G C A G T A C G T C G T A C G T A A C G T A C T G C T G A | Nkx6.1(Homeobox)/Islet-Nkx6.1-ChIP-Seq(GSE40975)/Homer | 1e-3 | -8.135e+00 | 0.0017 | 792.0 | 50.03% | 20970.7 | 45.69% | motif file (matrix) | svg |
| 76 | C G A T T A C G T G A C G A C T C A T G C G T A T A C G A C G T G T A C C T G A | Bach2(bZIP)/OCILy7-Bach2-ChIP-Seq(GSE44420)/Homer | 1e-3 | -8.054e+00 | 0.0018 | 62.0 | 3.92% | 1127.6 | 2.46% | motif file (matrix) | svg |
| 77 | T C G A G C A T A C G T C T A G G T A C T C G A G C A T T G A C T C G A A C G T | Chop(bZIP)/MEF-Chop-ChIP-Seq(GSE35681)/Homer | 1e-3 | -7.770e+00 | 0.0024 | 76.0 | 4.80% | 1468.7 | 3.20% | motif file (matrix) | svg |
| 78 | T A C G T C G A C A G T A C T G G C T A A T G C C G A T G T A C C G T A A C T G T A G C C G T A | NF-E2(bZIP)/K562-NFE2-ChIP-Seq(GSE31477)/Homer | 1e-3 | -7.721e+00 | 0.0025 | 27.0 | 1.71% | 378.8 | 0.83% | motif file (matrix) | svg |
| 79 | A G C T A C G T A C T G A T G C A G T C C G T A C T G A T A C G | NF1-halfsite(CTF)/LNCaP-NF1-ChIP-Seq(Unpublished)/Homer | 1e-3 | -7.572e+00 | 0.0029 | 408.0 | 25.77% | 10215.0 | 22.26% | motif file (matrix) | svg |
| 80 | G A C T C T A G A T G C A G T C G T C A T A C G A T G C A T C G | HIC1(Zf)/Treg-ZBTB29-ChIP-Seq(GSE99889)/Homer | 1e-3 | -7.485e+00 | 0.0031 | 448.0 | 28.30% | 11332.1 | 24.69% | motif file (matrix) | svg |
| 81 | A T G C G A T C G A C T A G C T C G A T C G A T G T C A C G A T T C G A A T C G T A G C T A G C | TATA-Box(TBP)/Promoter/Homer | 1e-3 | -7.447e+00 | 0.0032 | 391.0 | 24.70% | 9764.1 | 21.27% | motif file (matrix) | svg |
| 82 | A T G C A T C G T A C G A G C T A T C G C T G A A G T C C T A G A G C T A T G C C T G A A T G C | CRE(bZIP)/Promoter/Homer | 1e-3 | -7.158e+00 | 0.0042 | 50.0 | 3.16% | 892.6 | 1.94% | motif file (matrix) | svg |
| 83 | T C A G A T C G G A C T A C T G G A C T C A G T C T A G C G T A G T A C C G T A C T A G A T C G | Tbx20(T-box)/Heart-Tbx20-ChIP-Seq(GSE29636)/Homer | 1e-3 | -7.050e+00 | 0.0046 | 65.0 | 4.11% | 1245.2 | 2.71% | motif file (matrix) | svg |
| 84 | T C A G T A G C G A C T C A T G C T G A A T C G G C A T G T A C C G T A A C T G T A G C T G C A | MafK(bZIP)/C2C12-MafK-ChIP-Seq(GSE36030)/Homer | 1e-3 | -7.006e+00 | 0.0047 | 78.0 | 4.93% | 1558.2 | 3.39% | motif file (matrix) | svg |
| 85 | C A T G A C T G C T A G T C G A T C G A T C G A T C G A T C A G T C A G T C A G T G A C T G A C C G T A A C T G T G C A C G A T A C T G | RBPJ:Ebox(?,bHLH)/Panc1-Rbpj1-ChIP-Seq(GSE47459)/Homer | 1e-3 | -6.924e+00 | 0.0051 | 91.0 | 5.75% | 1880.5 | 4.10% | motif file (matrix) | svg |
| 86 | A G T C A T C G C T A G A G C T G A C T C T A G A G T C A G T C G C T A C A G T T C A G T C A G G A T C C T G A T C G A G A T C | RFX(HTH)/K562-RFX3-ChIP-Seq(SRA012198)/Homer | 1e-2 | -6.846e+00 | 0.0054 | 26.0 | 1.64% | 381.7 | 0.83% | motif file (matrix) | svg |
| 87 | T C G A A C T G A C T G C G T A C G T A T C G A A G T C C T G A A T C G G T A C G C A T C A T G | ETS:E-box(ETS,bHLH)/HPC7-Scl-ChIP-Seq(GSE22178)/Homer | 1e-2 | -6.779e+00 | 0.0058 | 30.0 | 1.90% | 466.2 | 1.02% | motif file (matrix) | svg |
| 88 | T A C G T A G C G C T A C G A T C T A G A C G T C A G T C A G T G C T A A G T C G T C A G C A T | FOXK2(Forkhead)/U2OS-FOXK2-ChIP-Seq(E-MTAB-2204)/Homer | 1e-2 | -6.760e+00 | 0.0058 | 199.0 | 12.57% | 4663.2 | 10.16% | motif file (matrix) | svg |
| 89 | A T C G A T C G A T G C G A C T T C G A C G T A G C A T A G C T C T G A T A C G | DLX2(Homeobox)/BasalGanglia-Dlx2-ChIP-seq(GSE124936)/Homer | 1e-2 | -6.688e+00 | 0.0062 | 493.0 | 31.14% | 12702.7 | 27.68% | motif file (matrix) | svg |
| 90 | C T A G C A T G C A T G T A C G A G T C G C A T A G C T C T A G A C G T A G T C G A C T A C T G A C T G A C T G T C G A | Zfp809(Zf)/ES-Zfp809-ChIP-Seq(GSE70799)/Homer | 1e-2 | -6.631e+00 | 0.0064 | 36.0 | 2.27% | 600.2 | 1.31% | motif file (matrix) | svg |
| 91 | T G C A T C G A T C G A A C G T C A T G C G T A A G T C T C A G A C G T G T A C C G T A A G C T | CREB5(bZIP)/LNCaP-CREB5.V5-ChIP-Seq(GSE137775)/Homer | 1e-2 | -6.607e+00 | 0.0065 | 114.0 | 7.20% | 2476.1 | 5.39% | motif file (matrix) | svg |
| 92 | A G T C G C A T C G T A C G T A G T A C A C G T A C T G G A T C G A T C T C G A | BMYB(HTH)/Hela-BMYB-ChIP-Seq(GSE27030)/Homer | 1e-2 | -6.518e+00 | 0.0071 | 395.0 | 24.95% | 10002.9 | 21.79% | motif file (matrix) | svg |
| 93 | C T A G T A C G G A T C G T C A T C G A A C G T T C A G C G T A C G T A C G T A | Hoxd10(Homeobox)/ChickenMSG-Hoxd10.Flag-ChIP-Seq(GSE86088)/Homer | 1e-2 | -6.298e+00 | 0.0087 | 374.0 | 23.63% | 9454.4 | 20.60% | motif file (matrix) | svg |
| 94 | C G T A A C G T A C G T A C G T A C G T A G T C A G T C C T G A A G C T A G C T | NFAT(RHD)/Jurkat-NFATC1-ChIP-Seq(Jolma\_et\_al.)/Homer | 1e-2 | -6.220e+00 | 0.0093 | 278.0 | 17.56% | 6839.3 | 14.90% | motif file (matrix) | svg |
| 95 | G T A C G A T C C A G T A G T C A G T C A G T C T G C A G A T C C T G A A T G C G T C A A C G T | WT1(Zf)/Kidney-WT1-ChIP-Seq(GSE90016)/Homer | 1e-2 | -6.150e+00 | 0.0099 | 104.0 | 6.57% | 2258.0 | 4.92% | motif file (matrix) | svg |
| 96 | G C A T A T C G C A T G G T A C G C T A A G T C T C A G T G A C G T C A T G C A | Arnt:Ahr(bHLH)/MCF7-Arnt-ChIP-Seq(Lo\_et\_al.)/Homer | 1e-2 | -6.093e+00 | 0.0104 | 154.0 | 9.73% | 3548.8 | 7.73% | motif file (matrix) | svg |
| 97 | T G C A C T G A A G T C G T C A A C T G A C T G C G T A C G T A C T G A A G C T | EWS:FLI1-fusion(ETS)/SK\_N\_MC-EWS:FLI1-ChIP-Seq(SRA014231)/Homer | 1e-2 | -6.079e+00 | 0.0104 | 149.0 | 9.41% | 3419.0 | 7.45% | motif file (matrix) | svg |
| 98 | G T C A G C A T A C T G G T A C G A C T A C T G G C T A A T C G C A G T G T A C C G T A A G C T | Nrf2(bZIP)/Lymphoblast-Nrf2-ChIP-Seq(GSE37589)/Homer | 1e-2 | -6.020e+00 | 0.0109 | 22.0 | 1.39% | 322.7 | 0.70% | motif file (matrix) | svg |
| 99 | C T G A C T G A C A T G A T C G A G C T A T C G G A C T C A T G C T G A G T C A | Tbr1(T-box)/Cortex-Tbr1-ChIP-Seq(GSE71384)/Homer | 1e-2 | -5.973e+00 | 0.0113 | 370.0 | 23.37% | 9392.0 | 20.46% | motif file (matrix) | svg |
| 100 | T C G A G C A T A C T G C T G A A G T C T C A G G A C T G T A C C G T A A G C T A G T C G A T C | c-Jun-CRE(bZIP)/K562-cJun-ChIP-Seq(GSE31477)/Homer | 1e-2 | -5.711e+00 | 0.0146 | 76.0 | 4.80% | 1589.0 | 3.46% | motif file (matrix) | svg |
| 101 | A T G C G A C T A C G T C T A G A C G T A C G T A C G T C T G A G A T C G C T A A G C T C G T A | Foxa2(Forkhead)/Liver-Foxa2-ChIP-Seq(GSE25694)/Homer | 1e-2 | -5.683e+00 | 0.0148 | 248.0 | 15.67% | 6095.3 | 13.28% | motif file (matrix) | svg |
| 102 | T C A G G C T A T C A G C A G T T G A C G T C A A G T C A T C G T G C A G T A C C A G T G A T C | Npas4(bHLH)/Neuron-Npas4-ChIP-Seq(GSE127793)/Homer | 1e-2 | -5.530e+00 | 0.0171 | 160.0 | 10.11% | 3761.3 | 8.19% | motif file (matrix) | svg |
| 103 | C T G A A T G C C G T A A C G T A G T C A G T C A C G T A C T G A T C G G C A T | SPDEF(ETS)/VCaP-SPDEF-ChIP-Seq(SRA014231)/Homer | 1e-2 | -5.432e+00 | 0.0187 | 265.0 | 16.74% | 6589.5 | 14.36% | motif file (matrix) | svg |
| 104 | T C G A C T G A T A G C T G A C T C A G T C A G C G T A C G T A T C A G A G C T | ETV1(ETS)/GIST48-ETV1-ChIP-Seq(GSE22441)/Homer | 1e-2 | -5.417e+00 | 0.0188 | 317.0 | 20.03% | 8013.4 | 17.46% | motif file (matrix) | svg |
| 105 | C T A G T A C G G A C T T G C A T G C A C G A T T A C G C T G A T C G A C T G A | Hoxa10(Homeobox)/ChickenMSG-Hoxa10.Flag-ChIP-Seq(GSE86088)/Homer | 1e-2 | -5.335e+00 | 0.0202 | 191.0 | 12.07% | 4605.4 | 10.03% | motif file (matrix) | svg |
| 106 | A T G C T C A G T C G A G C A T A C T G C G T A A G T C T C A G G A C T T G A C C G T A A G C T | Atf2(bZIP)/3T3L1-Atf2-ChIP-Seq(GSE56872)/Homer | 1e-2 | -5.174e+00 | 0.0235 | 79.0 | 4.99% | 1701.9 | 3.71% | motif file (matrix) | svg |
| 107 | C T G A C G A T C T A G T C A G G A T C C T G A T C A G G A T C C T G A A C T G A G T C G C T A A C G T A G T C G C A T | PRDM9(Zf)/Testis-DMC1-ChIP-Seq(GSE35498)/Homer | 1e-2 | -5.086e+00 | 0.0254 | 113.0 | 7.14% | 2576.3 | 5.61% | motif file (matrix) | svg |
| 108 | C T A G T A C G G A T C G T C A T G C A A C G T T G C A G C T A T C G A T G C A | Hoxa9(Homeobox)/ChickenMSG-Hoxa9.Flag-ChIP-Seq(GSE86088)/Homer | 1e-2 | -4.989e+00 | 0.0277 | 778.0 | 49.15% | 21123.6 | 46.02% | motif file (matrix) | svg |
| 109 | T G C A C T G A A T G C G T C A A C T G A C T G C G T A C G T A C T A G A G C T | Ets1-distal(ETS)/CD4+-PolII-ChIP-Seq(Barski\_et\_al.)/Homer | 1e-2 | -4.960e+00 | 0.0283 | 80.0 | 5.05% | 1742.9 | 3.80% | motif file (matrix) | svg |
| 110 | T C A G T A C G T A G C A C G T A C T G C G A T A G T C C G T A T A C G A G T C | Meis1(Homeobox)/MastCells-Meis1-ChIP-Seq(GSE48085)/Homer | 1e-2 | -4.891e+00 | 0.0300 | 405.0 | 25.58% | 10537.0 | 22.96% | motif file (matrix) | svg |
| 111 | C G T A C T A G T C A G T C A G A G T C A T G C A G T C G C A T A G C T A C G T A T C G C G A T | Sox9(HMG)/Limb-SOX9-ChIP-Seq(GSE73225)/Homer | 1e-2 | -4.789e+00 | 0.0330 | 241.0 | 15.22% | 6024.4 | 13.13% | motif file (matrix) | svg |
| 112 | A T G C T A C G A G T C G A C T T G C A C T G A G A C T A C G T C T G A T C A G | LHX9(Homeobox)/Hct116-LHX9.V5-ChIP-Seq(GSE116822)/Homer | 1e-2 | -4.766e+00 | 0.0335 | 438.0 | 27.67% | 11482.8 | 25.02% | motif file (matrix) | svg |
| 113 | C T A G A G C T G A C T C A T G A G T C A G T C G T C A C A G T C T A G T C A G G T A C C T G A T C G A G A T C T G A C | Rfx2(HTH)/LoVo-RFX2-ChIP-Seq(GSE49402)/Homer | 1e-2 | -4.717e+00 | 0.0348 | 26.0 | 1.64% | 450.3 | 0.98% | motif file (matrix) | svg |
| 114 | C G A T C T A G T C G A A G C T C G A T C T G A C G T A A G C T A C T G C T A G A T G C G A T C | Hoxb4(Homeobox)/ES-Hoxb4-ChIP-Seq(GSE34014)/Homer | 1e-2 | -4.693e+00 | 0.0353 | 63.0 | 3.98% | 1335.4 | 2.91% | motif file (matrix) | svg |
| 115 | T G A C G T C A A G T C G C T A C T A G A G T C C T G A C A T G A C T G C T A G T A C G T C A G | Zic2(Zf)/ESC-Zic2-ChIP-Seq(SRP197560)/Homer | 1e-2 | -4.674e+00 | 0.0357 | 86.0 | 5.43% | 1917.1 | 4.18% | motif file (matrix) | svg |
| 116 | T A C G T C G A G A C T A C T G C T G A A G T C T C A G G A C T T G A C C T G A | Atf1(bZIP)/K562-ATF1-ChIP-Seq(GSE31477)/Homer | 1e-2 | -4.664e+00 | 0.0358 | 165.0 | 10.42% | 3989.6 | 8.69% | motif file (matrix) | svg |
| 117 | G A C T C T A G C T A G C T A G A C T G T C G A C T G A C T A G C T A G C T A G G T A C G T C A | ZNF467(Zf)/HEK293-ZNF467.GFP-ChIP-Seq(GSE58341)/Homer | 1e-2 | -4.655e+00 | 0.0358 | 156.0 | 9.85% | 3750.5 | 8.17% | motif file (matrix) | svg |
| 118 | T G C A G C A T C G A T C G T A C A G T A C T G G T A C C G T A C T G A A G C T G T C A A C T G C T A G G T C A C G A T A C T G G T A C T G C A C G T A A G C T | CEBP:CEBP(bZIP)/MEF-Chop-ChIP-Seq(GSE35681)/Homer | 1e-2 | -4.649e+00 | 0.0358 | 51.0 | 3.22% | 1042.8 | 2.27% | motif file (matrix) | svg |
